# Risk of cancer development in patients with keloids

**DOI:** 10.1038/s41598-021-88789-1

**Published:** 2021-04-30

**Authors:** Ying-Yi Lu, Hung-Pin Tu, Chieh-Hsin Wu, Chien-Hui Hong, Kuo-Chia Yang, Hui-Ju Yang, Kee-Lung Chang, Chih-Hung Lee

**Affiliations:** 1 Department of Dermatology, Kaohsiung Veterans General Hospital, Kaohsiung, Taiwan; 2 Graduate Institute of Medicine, College of Medicine, Kaohsiung Medical University, No. 100, Shih-Chuan 1st Road, Kaohsiung, 80708 Taiwan; 3 Shu-Zen Junior College of Medicine and Management, Kaohsiung, Taiwan; 4 Department of Public Health and Environmental Medicine, School of Medicine, College of Medicine, Kaohsiung Medical University, Kaohsiung, Taiwan; 5 Division of Neurosurgery, Department of Surgery, Kaohsiung Medical University Hospital, Kaohsiung, Taiwan; 6 Department of Surgery, School of Medicine, College of Medicine, Kaohsiung Medical University, Kaohsiung, Taiwan; 7 Department of Dermatology, Faculty of Medicine, National Yang Ming Chiao Tung University, Taipei, Taiwan; 8 Departments of Dermatology, Changhua Christian Hospital, No. 135, Nanxiao St., Changhua City, Changhua County 500054 Taiwan; 9 Department of Biochemistry, School of Medicine, College of Medicine, Kaohsiung Medical University, Kaohsiung, Taiwan; 10 Department of Medical Research, Kaohsiung Medical University Hospital, Kaohsiung, Taiwan; 11 Department of Dermatology, Kaohsiung Chang Gung Memorial Hospital and Chang Gung University College of Medicine, No. 123, Ta Pei Rd., Niaosong Dist., Kaohsiung, 833 Taiwan; 12 Institute of Medicine, Chung Shan Medical University, Taichung, Taiwan

**Keywords:** Cancer, Diseases, Medical research, Risk factors

## Abstract

Keloid is a skin disease characterized by exaggerated scar formation, excessive fibroblast proliferation, and excessive collagen deposition. Cancers commonly arise from a fibrotic microenvironment; e.g., hepatoma arises from liver cirrhosis, and oral cancers arise from submucosal fibrosis. As keloids are a prototypic fibroproliferative disease, this study investigated whether patients with keloids have an increased cancer risk. In a matched, population-based study, first 17,401 patients treated for keloids during 1998–2010 with 69,604 controls without keloids at a ratio of 1:4 were evaluated. The association between keloids and risk of cancer was estimated by logistic regression or Cox proportional hazard regression models after adjustment of covariates. In total, 893 first-time cases of cancer were identified in the 17,401 patients with keloids. The overall cancer risk was 1.49-fold higher in the keloids group compared to controls. Regarding specific cancers, the keloids group, had a significantly higher risk of skin cancer compared to controls (Relative risk = 1.73). The relative risk for skin cancer was even higher for males with keloids (Relative risk = 2.16). Further stratified analyses also revealed a significantly higher risk of developing pancreatic cancer in female patients with keloids compared to controls (Relative risk = 2.19) after adjustment for known pancreatic cancer risk factors. This study indicates that patients with keloids have a higher than normal risk for several cancer types, especially skin cancers (both genders) and pancreatic cancer (females). Therefore, patients with keloids should undergo regular skin examinations, and females with keloids should regularly undergo abdominal ultrasonography.

## Introduction

Keloids are benign dermal fibro-proliferative tumors characterized by overproliferation of fibroblasts and excessive accumulation of collagen^1^. Keloids are triggered by cutaneous injury followed by abnormal wound recovery^2^. They often invade neighboring normal skin without regression^3^. They often cause intense itching and pain and usually recur after various treatments. Dark-skinned individuals are more susceptible to keloids compared to light-skinned individuals^4^. The exact etiology of keloids remains unclear but is apparently multifactorial. Genetic and environmental factors are often implicated^5,6^.

Cancer is the main cause of mortality worldwide. Uncontrolled fibrocyte proliferation and collagen deposition in keloids are analogous to uncontrolled cell growth and proliferation in cancers. Furthermore, many cancers develop from sclerotic and fibrous microenvironments, e.g., hepatoma develops from liver cirrhosis, oral cancer develops from submucosal fibrosis, and Majorlin’s cancer develops from burn scars. Since keloids are a prototypic fibrous proliferative and sclerotic disease of the skin, this study investigated whether patients with keloids have a higher than normal tendency to develop cancers, including skin and internal cancers. Notably, sporadic case reports show that keloids may be a paraneoplastic phenomenon^7–9^. Coppa et al. reported a 66-year-old woman who developed eruptive keloids but was diagnosed with endometrial carcinoma^9^. In He et al., an 81-year-old woman developed right breast cancer and eruptive keloids with severe pruritus over a roughly similar time period^7^. Sakaguchi et al. described the development of bilateral breast cancer in a 72-year-old woman with extensive keloids on the limbs, presternal area, and bilateral breasts. Notably, her serum level of transforming growth factor-β1 (TGF-β1) was abnormally high ^8^.

To date, no large epidemiological studies have investigated whether patients with keloids tend to develop cancers. Therefore, this retrospective study used the Taiwan National Health Insurance Research Database (NHIRD) to perform the first matched study in an Asian population.

## Results

### Demographic characteristics of the subjects (Table 1)

**Table 1:** The demographic characteristics of the keloids group and the control group.

Variables	Keloids		P value
	Yes	No	
	N = 17,401	N = 69,604	
Cancer, n (%)	893 (5.13)	2434 (3.50)	< 0.0001
Age mean (SD) (years)	38.4 (14.4)	38.4 (14.3)	0.7993
**Age group, n (%)**			
20–40	11,321 (65.1)	45,273 (65.0)	
41–60	4472 (25.7)	18,112 (26.0)	
> 60	1608 (9.2)	6219 (8.9)	0.3669
**Gender**			
Males	6635 (38.1)	26,540 (38.1)	
Females	10,766 (61.9)	43,064 (61.9)	1.0000
**Region, n (%)**			
Northern	7511 (43.2)	29,660 (42.6)	
Central	4759 (27.3)	19,156 (27.5)	
Southern	4553 (26.2)	18,215 (26.2)	
Eastern, Offshore islets, and other	578 (3.3)	2573 (3.7)	0.0878

*SD* standard deviation.

The analysis included 17,401 patients with keloids and 69,604 controls. The average age (38.4 years), gender distribution (61.9% female), and geographic distribution of the population were comparable after the adjustment strategy was applied (northern 43.2%, central 27.3%, southern 26.2%, eastern, offshore and other 3.3%). Keloids were slightly more common in females (61.9%) than in males. The majority of patients with keloids developed the disease at age 20–40 years old (65.1%), followed by 41–60 years old (25.7%) and > 60 years old (9.2%). In total, 893 (5.13%) patients developed cancer out of the 17,401 patients who had keloids but no prior cancers. In contrast, the 69,604 controls without keloids included 2434 patients who developed cancer (3.5%).

### Increased overall cancer risk in patients with keloids (Table 2)

**Table 2:** Total cancer risk in patients with keloids, stratified by gender.

Variables	Keloids	Non-keloids group	OR (95% CI)	P value
Overall cancers	893	2434	1.49 (1.38–1.61)	< 0.0001
**Gender**				
Males	332	871	1.55 (1.36–1.77)	< 0.0001
Females	561	1563	1.46 (1.32–1.61)	< 0.0001

*OR* odd ratio, *95% CI* 95% confidence interval.

To determine whether overall cancer risk was higher than normal in patients with keloids, logistic regression analysis was performed to evaluate the association between keloids and overall cancers. The results showed that, compared to controls, patients with keloids had a significantly higher odd ratio (OR) for developing cancer, which is 1.49 with 95% confidence interval (CI) 1.38–1.61. Additionally, the cancer risk was increased in both male and female patients with keloids.

### OR for specific cancers in patients with keloids (Table 3)

**Table 3:** Odd ratio of specific cancers in patients with keloids, stratified by gender.

Variables	All		Males		Females	
Cancer	OR (95% CI)	P value	OR (95% CI)	P value	OR (95% CI)	P value
Oral cancer	1.16 (0.92–1.46)	0.2162	1.19 (0.87–1.63)	0.2828	1.12 (0.79–1.59)	0.5099
Esophageal cancer	1.70 (1.06–2.71)	0.0270	1.91 (0.90–4.05)	0.0933	1.58 (0.87–2.87)	0.1340
Stomach cancer	1.29 (0.84–1.97)	0.2443	1.39 (0.79–2.46)	0.2547	1.17 (0.62–2.23)	0.6308
Colon cancer	1.54 (1.28–1.86)	< 0.0001	1.63 (1.24–2.15)	0.0005	1.47 (1.14–1.90)	0.0027
Liver cancer	1.59 (1.30–1.95)	< 0.0001	1.82 (1.37–2.43)	< 0.0001	1.40 (1.05–1.87)	0.0212
Pancreatic cancer	2.57 (1.59–4.18)	0.0001	2.20 (1.05–4.60)	0.0356	2.91 (1.53–5.55)	0.0011
Lung cancer	1.12 (0.85–1.46)	0.4274	1.06 (0.73–1.54)	0.7564	1.18 (0.80–1.74)	0.4061
Melanoma	3.40 (1.78–6.50)	0.0002	3.20 (1.26–8.12)	0.0142	3.60 (1.46–8.87)	0.0053
Skin cancer	4.35 (3.16–5.99)	< 0.0001	5.89 (3.64–9.53)	< 0.0001	3.37 (2.18–5.22)	< 0.0001
Kaposi’s sarcoma	2.00 (0.18–22.06)	0.5714	4.00 (0.25–63.97)	0.3269	–	
Breast cancer	–		–		1.28 (1.03–1.58)	0.0230
Cervical cancer	–		–		1.09 (0.78–1.53)	0.5998
Uterine cancer	–		–		1.24 (0.78–1.99)	0.3611
Ovarian cancer	–		–		1.32 (0.93–1.85)	0.1156
Prostate cancer	–		1.63 (1.14–2.32)	0.0073	–	
Bladder cancer	1.16 (0.72–1.86)	0.5445	1.04 (0.57–1.92)	0.8985	1.38 (0.65–2.96)	0.3999
Kidney cancer	1.77 (1.03–3.03)	0.0386	1.69 (0.74–3.85)	0.2159	1.83 (0.90–3.75)	0.0958
Unspecified urinary organ cancer	0.87 (0.44–1.72)	0.6887	0.44 (0.10–1.92)	0.2765	1.14 (0.52–2.51)	0.7390
Thyroid cancer	1.71 (1.22–2.41)	0.0021	0.86 (0.25–2.98)	0.8085	1.84 (1.28–2.63)	0.0009
Hodgkin’s lymphoma	1.77 (1.13, 2.79)	0.0134	3.00 (1.04–8.65)	0.0419	0.67 (0.08–5.54)	0.7074
Non-Hodgkin’s lymphoma	2.00 (0.81–4.96)	0.1342	1.29 (0.63–2.63)	0.4827	2.27 (1.25–4.12)	0.0070

*OR* odd ratio, *95% CI* 95% confidence interval.

After identifying an increased overall cancer risk in patients with keloids, we next investigated what specific cancers are associated with keloids. The analysis showed that, compared to controls, the patients with keloids had an increased association with esophageal cancer (OR = 1.7), colon cancer (OR = 1.54), liver cancer (OR = 1.59), pancreatic cancer (OR = 2.57), melanoma (OR = 3.4), nonmelanoma skin cancers (OR = 4.35), breast cancer (OR = 1.28), prostate cancer (OR = 1.63), kidney cancer (OR = 1.77), thyroid cancer (OR = 1.71), and Hodgkin’s lymphoma (OR = 1.77). Additionally, female patients with keloids were associated with non-Hodgkin’s lymphoma (OR = 2.27), as well.

### Adjusted RR of specific cancers in patients with keloids (Table 4)

**Table 4:** Relative risk of specific cancers in patients with keloids, stratified by gender.

Variables	All				Males				Females			
Cancer	Crude RR (95% CI)	P value	Adjusted RR^a^ (95% CI)	P value	Crude RR (95% CI)	P value	Adjusted RR^a^ (95% CI)	P value	Crude RR (95% CI)	P value	Adjusted RR^a^ (95% CI)	P value
Esophageal cancer	0.68 (0.35–1.33)	0.2575	0.67 (0.34–1.30)	0.2337								
Colon cancer	0.68 (0.53–0.88)	0.0031	0.66 (0.51–0.86)	0.0016	0.71 (0.48–1.03)	0.0728	0.66 (0.45–0.96)	0.0316	0.66 (0.47–0.93)	0.0184	0.66 (0.47–0.94)	0.0196
Liver cancer	0.74 (0.57–0.98)	0.0327	0.73 (0.56–0.96)	0.0251	0.83 (0.56–1.22)	0.3491	0.80 (0.54–1.17)	0.2527	0.67 (0.45–0.98)	0.0416	0.67 (0.46–0.99)	0.0440
Pancreatic cancer	1.72 (0.99–2.98)	0.0555	1.66 (0.95–2.88)	0.0730	1.20 (0.48–2.99)	0.6940	1.11 (0.44–2.76)	0.8295	2.18 (1.08–4.41)	0.0296	2.19 (1.08–4.42)	0.0293
Melanoma	1.40 (0.59–3.31)	0.4424	1.39 (0.59–3.29)	0.4554	1.60 (0.50–5.10)	0.4263	1.47 (0.46–4.71)	0.5130	1.20 (0.33–4.36)	0.7811	1.25 (0.34–4.55)	0.7338
Skin cancer	1.73 (1.13–2.63)	0.0110	1.73 (1.13–2.63)	0.0112	2.29 (1.24–4.24)	0.0081	2.16 (1.17–4.00)	0.0139	1.37 (0.76–2.46)	0.2961	1.43 (0.79–2.57)	0.2329
Breast cancer									0.58 (0.43–0.78)	0.0003	0.58 (0.43–0.78)	0.0003
Prostate cancer					0.57 (0.33–0.98)	0.0406	0.48 (0.28–0.83)	0.0083				
Kidney cancer	0.65 (0.29–1.45)	0.2933	0.66 (0.30–1.47)	0.3116								
Thyroid cancer	0.76 (0.48–1.22)	0.2604	0.77 (0.48–1.23)	0.2690					0.79 (0.49–1.3)	0.3571	0.79 (0.49–1.3)	0.3591
Hodgkin’s lymphoma					0.50 (0.06–4.00)	0.5139	0.51 (0.06–4.07)	0.5242				
Non-Hodgkin’s lymphoma	0.92 (0.51–1.64)	0.7748	0.92 (0.51–1.64)	0.7781					1.47 (0.74–2.93)	0.2759	1.48 (0.74–2.95)	0.2662

*RR* relative risk, *95% CI* 95% confidence interval.

^a^Model adjusted for age group, gender, and the residential region using a Cox proportional-hazards regression model.

We then focused on the specific cancers that showed increases in OR. A cohort study was then performed to evaluate the association between keloids and specific cancer types. From January 1, 1998 to December 31, 2010, in patients who had been diagnosed with specific cancer types before their diagnosis of keloids, temporality was corrected, and the patients were excluded. The results showed that keloids patients had increased risk of skin cancer (RR = 1.73, 95% CI 1.13–2.63). Surprisingly, however, keloids patients had lower risks of colon cancer (RR = 0.66, 95% CI 0.51–0.86) and liver cancer (RR = 0.73, 95% CI 0.56–0.96). Further gender-stratified analysis revealed that male patients with keloids were vulnerable to skin cancer (RR = 2.16, 95% CI 1.17–4.00) but protected from prostate cancer (RR = 0.48, 95% CI 0.28–0.83) or colon cancer (RR = 0.66, 95% CI 0.45–0.96). Female patients with keloids were vulnerable to pancreatic cancer (RR = 2.39, P = 0.0293) but protected from breast cancer (RR = 0.55, 95% CI = 0.43–0.78), colon cancer (RR = 0.66, 95% CI 0.47–0.94) or liver cancer (RR = 0.67, 95% CI 10.46–0.99). Besides, female patients with keloids had an increased risk of skin cancer although this risk does not reach statistical significance.

### Keloids were an independent risk factor for pancreatic cancer in females

The incidence of skin cancers was consistently higher in patients with keloids compared to controls. Female patients with keloids had an increased risk of pancreatic cancer (RR = 2.19, 95% CI 1.08–4.42). Previous studies have found that chronic pancreatitis, liver cirrhosis, and diabetes mellitus are important risk factors for pancreatic cancer ^10,11^. Therefore, RRs were calculated after adjustment for these covariates and stratification by these comorbidities (Table 5). While chronic pancreatitis was the most important risk factor for pancreatic cancer (RR = 16.71), the keloids group still had a higher risk of developing pancreatic cancer compared to controls (RR = 2.05). Next, Cox proportional hazard regression analysis was used to investigate the interacting effects of chronic pancreatitis and keloids on the development of pancreatic cancers. Table 6 shows that chronic pancreatitis and keloids did not have a statistically significant interacting effect (p for interaction 0.5695). The data indicated that keloids and chronic pancreatitis were both independent risk factors and joint risk factors for pancreatic cancers.

**Table 5:** Pancreatic cancer risk in female patients with keloids, stratified by comorbidities.

Variables	N	Pancreatic cancer	Crude RR (95% CI)	P-value	Adjusted RR^a^ (95% CI)	P-value
Non-keloids group	43,064	22	1.00		1.00	
Keloids	10,762	12	2.18 (1.08–4.41)	0.0296	2.05 (1.01–4.17)	0.0459
**Liver cirrhosis**						
No	53,488	33	1.00		1.00	
Yes	338	1	4.80 (0.66–35.06)	0.1224	1.53 (0.2–11.5)	0.6772
**Chronic pancreatitis**						
No	53,707	31	1.00		1.00	
Yes	119	3	43.68 (13.35–142.86)	< 0.0001	16.71 (4.91–56.87)	< 0.0001
**Diabetes mellitus**						
No	48,539	24	1.00		1.00	
Yes	5287	10	3.83 (1.83–8.00)	0.0004	1.22 (0.54–2.73)	0.6362

*RR* relative risk, *95% CI* 95%confidence interval.

^a^Model adjusted for age group, gender, the residential region and related comorbidities using a Cox proportional-hazards regression model.

**Table 6:** Interacting effects of keloids and chronic pancreatitis on the development of pancreatic cancer in female patients with keloids.

Variables		N	Pancreatic cancer	Crude RR (95% CI)	P-value	Adjusted RR^a^ (95% CI)	P-value	P for interaction^b^
Keloids	Chronic pancreatitis							0.5695
No	No	42,979	20	Reference		Reference		
Yes	No	10,728	11	2.20 (1.06–4.60)	0.0353	2.20 (1.05–4.60)	0.0355	
No	Yes	85	2	50.56 (11.82–216.33)	< 0.0001	22.34(5.06–98.65)	< 0.0001	
Yes	Yes	34	1	63.22 (8.49–470.95)	< 0.0001	23.67(3.10–180.78)	0.0023	

*RR* relative risk, *CI* confidence interval.

^a^Model adjusted for age group, gender, and the residential region, liver cirrhosis and diabetes mellitus using a Cox proportional-hazards regression model.

^b^Interactions between keloid cases and chronic pancreatitis were calculated using the Cox proportional hazards regression model with an added interaction term for keloid cases by chronic pancreatitis and covariates.

## Discussion

To the best of our knowledge, this study is the first to demonstrate an association between keloids and human cancers in an Asian population. The main findings of the study support our hypothesis that keloids patients have a higher than normal risk of developing cancers, especially skin cancer and pancreatic cancer. Overall cancer risk was 1.49-fold higher in the keloids group compared to the control group. Notably, the cancer risk in keloids patients was increased regardless of gender. The relationship between cancers and keloids varied by cancer type. Keloids patients had a 1.73-fold higher risk of developing skin cancer compared to non-keloids patients, and male patients with keloids had an even higher RR (2.16).

The underlying mechanisms of the association between keloids and human cancers remain to be determined. However, several lines of evidence from previous studies indicate that keloids and human cancers may share similar pathophysiological processes. Firstly, prolonged inflammation with elevated proinflammatory cytokines in injured tissues contributes to keloidogenesis^12–18^. Chronic inflammation is known to promote tumor development and progression in skin cancers, melanoma and pancreatic cancers^19^. For example, studies have shown that nuclear factor-kappa B, activator and signal transducer of transcription 3, hypoxia-inducible factor-1 alpha cytokines and chemokines play critical role of inflammation in the carcinogenesis of skin cancer and pancreatic cancer ^19,20^. Interestingly, patients with chronic pancreatitis are known to have a high risk of developing pancreatic cancer, which suggests that chronic inflammatory processes may be the master mediators of pancreatic cancer ^21^. In short, chronic inflammation is likely to result in fibrosis that may mediate the development of both keloids and cancers.

Secondly, TGF-β1 has emerged as a potent growth factor in wound healing and inflammation. The TGF-β1 regulates cell growth and differentiation as well as cancer initiation and progression. Many studies have reported that elevated TGF-β1 in keloidal tissue stimulates dermal fibroblast proliferation and differentiation as well as collagen formation^22^. The TGF-β regulates phosphorylation of Smad family proteins^23^. Wu el al. reported that TGF-β1 increases phosphorylation of Smad2/3 and Smad4 in keloidal fibroblasts^24^. Jialiang el al., which was the first study to demonstrate increased Smad4 expression and decreased Smad7 expression in keloids, proposed that Toll-like receptors (TLRs) exert contribute to keloidogenesis through TGF-β/Smad signaling^25^. Subsequent studies have confirmed the critical role of TGF-β/Smad signaling in keloidogenesis^26–31^. Similarly, TGF-β/Smad signaling reportedly has roles in the development of skin cancers and pancreatic cancer^32^. The TGF-β1 secreted from pancreatic damaged acinar cells and cancer cells is a fibrotic growth or pro-tumorigenic factor^33,34^. Therefore, TGF-β/Smad signaling has a pivotal role in the pathogenesis of keloids and cancers.

Finally, micro-RNAs (miRNAs) can contribute to cancer development. This group of conserved, non-coding, and single-stranded small RNA molecules of 21–25 nucleotides control gene expressions and are important for maintaining cellular hemostasis^35,36^. Specific miRNA dysregulation has profound impacts on cellular physiological changes that have been associated with various diseases^37^. The pleiotropic role of miRNAs in metastasis, fibrotic processes, and genesis of cancer is well-documented^38–41^. Reduced expression of some miRNAs, e.g., miR-29, has been associated with keloids development^42–46^. Similarly, cancer cells often exhibit reduced expression of miR-29 family members that have important roles in the tumor microenvironment and cancer pathogenesis ^47,48^. For example, Schmitt et al*.* reported that patients with metastatic melanoma had low expressions of miR-29a and miR-29b^49^, and Kwon et al. showed that decreased miR-29 in activated pancreatic stellate cells correlated with increased extracellular matrix^50^.

The incidence of pancreatic cancer is low, but it increases at a rate of 1.4–1.8% per year of age. The dismal 5-year survival rate (8%) results in high mortality^51^. Only two studies have reported an association between pancreatic tumors and keloids. Speer et al. analyzed a 20-year (1991–2011) series of pediatric patients treated for solid pseudopapillary tumor of the pancreas at a single institution. The authors reported that these patients had a high incidence of developing incisional keloids after abdominal surgery^52^. Pasquali et al*.* reported that only one patient developed keloids out of 109 patients who had undergone surgery for neuroendocrine pancreatic tumors during 1980–2001^53^. However, the association between pancreatic cancer and keloids requires further study. As mentioned above, the link between pancreatic cancer and keloids may be attributable to chronic inflammation and TGF-β/Smad signaling. The Akt is activated in both skin cancers and pancreatic cancers^54,55^. Clinical evidence also indicates that miR-21 and miR-29 share a common pathophysiology in the development of keloids and pancreatic cancers. Furthermore, YAP (yes-associated protein) and TAZ (transcriptional coactivator with PDZ-binding motif) are part of a classic pathway that controls the contact inhibition in the Hippo signaling pathway. They also have a similar role in the pathophysiology of keloids and pancreatic cancer development, i.e., modulation of pancreatic tissue regeneration, neoplastic transformation, and stellate cell function^56^.

The major strength in this study is the use of a database from a nationwide, single-payer health care system. The large sample size provided sufficient statistical power for comparisons. Moreover, since data were obtained from a nationwide registry rather than from a hospital-based registry, the calculation of cancer incidences was accurate. Another strength of this study is that the data were derived from the Taiwan nationwide healthcare system, in which most participants are Asians. Therefore, analysis of this population yields important insights into the roles of genetic ancestry in keloids and cancers.

Some limitations of this study are noted. First, for better ICD code accuracy, the occurrence of keloid was defined as diagnoses made by a dermatologist or a plastic surgeon in this study. The specificity of keloid diagnosis in this manner is high, however, the actual incidence of keloids may be underestimated. Second, the database did not provide information that could be used to determine how the severity, extent, or location of keloids affects cancer risk. Third, the database did not provide information about genetic and environmental factors that can have important roles in keloids pathogenesis as well as cancer formation; such factors include body mass index, lifestyle, social habits, family history, and laboratory examination data. Fourth, most participants were ethnic Chinese, which limits the ability to generalize the results to other racial populations. Finally, although statistically significant, further mechanistic studies are warranted to understand the pathomechanisms.

## Conclusions

In summary, the association between human cancers and keloids is organ specific. This study is the first to analyze cancer risk, specifically, pancreatic cancer risk in females and skin cancer risk in both genders, in an Asian population of patients with keloids. The results of this study indicate that, regardless of gender, patients with keloids should routinely undergo skin examination to ensure early identification of skin cancers. Additionally, female patients with keloids should undergo scheduled physical and laboratory follow-up examinations, e.g., abdominal ultrasonography and blood tumor marker test, to screen for pancreatic cancer.

## Materials and methods

### Data source

The NHIRD, one of the largest nationwide population-based databases, was established by the National Health Research Institutes in Taiwan. This encrypted secondary database implemented in March 1995, contains data for Taiwan residents covered by single-payer government insurance, which currently includes 99% of all Taiwan residents. The original medical claims data contained in the NHIRD are available to researchers and have been used extensively for epidemiological studies in Taiwan^57^. Data retrieved from the NHIRD was de-identified and encrypted. In accordance with Personal Electronic Data Protection Law, individual information which can be utilized to identify beneficiaries and hospitals must be removed from the NHIRD. All data from the NHIRD were anonymized to protect the participants’ privacy. This study used the NHIRD Longitudinal Health Insurance Database (LHID) 2010, which contains data for a nationally representative random sample of 1 million insurance beneficiaries who enrolled in the NHIRD in 2010.

### Study design

All diagnoses in the analysis were coded according to the International Classification of Diseases, Ninth Revision, Clinical Modification (ICD-9-CM). First, 17,401 patients with keloids (ICD-9-CM code 701.4) were identified in the LHID ^58,59^. A comparison group of 69,604 patients without keloids were then matched at a ratio of 1:4 for age, age group, gender, and region of residence by propensity scores.

**Figure 1:**
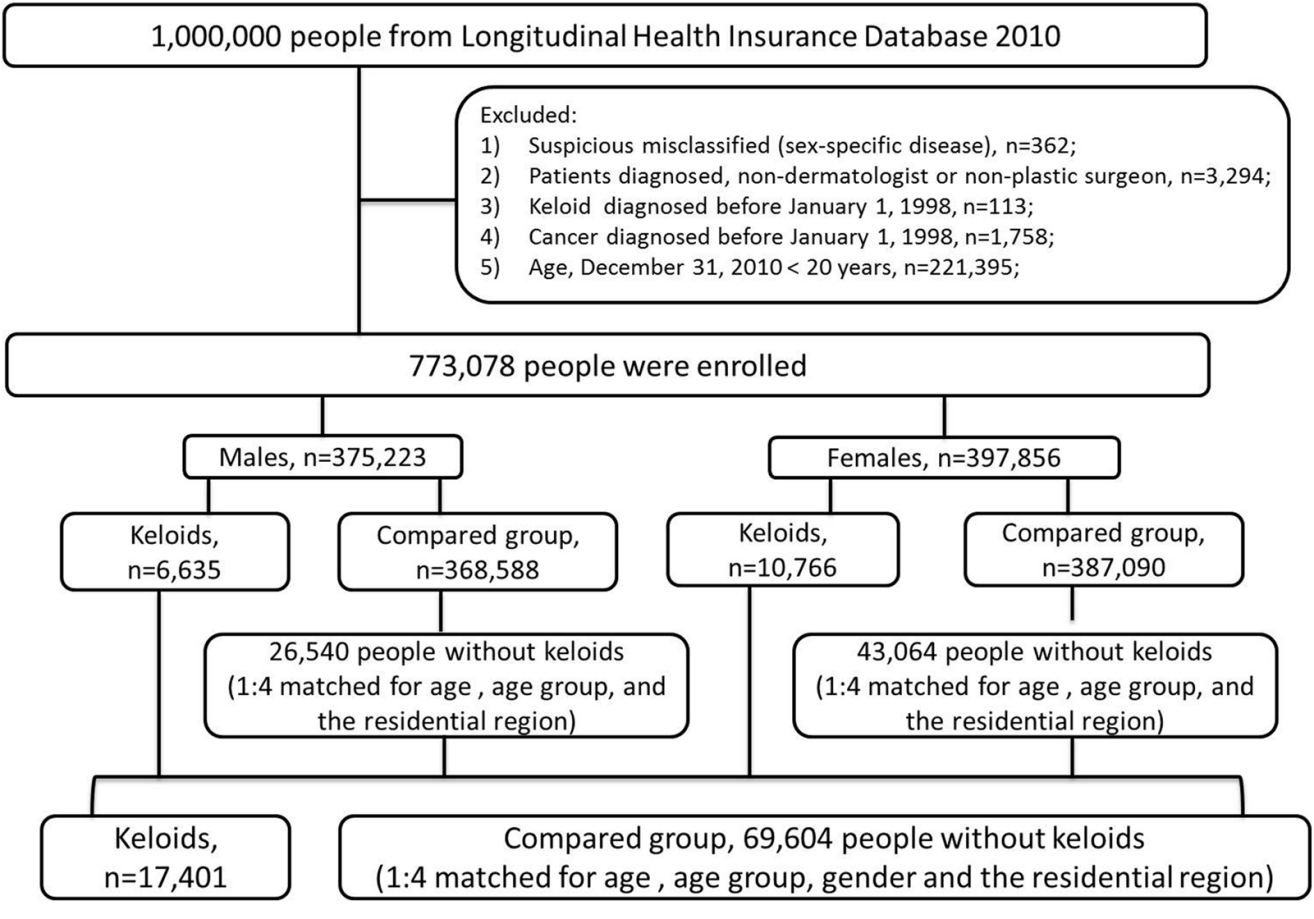
Workflow of the diagnostic process for keloids and cancers using the Longitudinal Health Insurance Database 2010.

Figure 1 shows the workflow of the study population. This is a population-based observational study with a cross-sectional and a retrospective cohort study design to elucidate the association between keloid and cancer. At first using a cross sectional design in the database of LHID2010, all patients with a record of a first-time diagnosis of keloids by a dermatologist or a plastic surgeon from January 1, 1998 to December 31, 2010 were eligible for inclusion in this study. When subjects with keloids were categorized by many different type of cancers, numbers of the subjects in each category (by cancer types) could be too low to distort the actual prevalence. Therefore, in this first part of cross-sectional study, a logistic regression was performed to explore the association of keloid with overall incident cancer and different kind of cancers.

After the association of ALL cancers with keloids were successfully established by the logistic regression, a retrospective cohort study was performed to evaluate the risk of the development of specific cancers in patients with keloid. To overcome the initially overlooked odd risks by using logistic regression, in patients who had a specific cancer diagnosis before a keloid diagnosis (the index date of keloid onset), temporality was corrected, and these patients were excluded. Relative risk of specific cancer was determined by Cox proportional hazard regression modeling after adjusting common covariates (age, gender and the residential region).

### Ethical approval

The study was performed according to Declaration of Helsinki guidelines and was approved by the Institutional Review Board of Chang Gung Medical Foundation. (IRB No. 201801614B1). According to the regulations of Institutional Review Board of Chang Gung Medical Foundation, the need for informed consent was waived per protocol.

### Measurement and definition

The occurrence of keloids was defined as ≥ 2 keloid diagnosis (ICD-9-CM code 701.4) in outpatient visits or inpatient visits. The index date of keloids onset was defined as the earliest date of a keloid diagnosis. Patients were excluded if they were younger than 20 years at the time of keloid diagnosis in 2010 in accordance to IRB regulations.

The primary outcome was overall incident cancer, including all the specific cancers as below. The secondary outcomes were different specific cancers: oral cancer (ICD-9-CM code 140-149), esophageal cancer (ICD-9-CM code 150), stomach cancer (ICD-9-CM code 151), colon cancer (ICD-9-CM code 153), liver cancer (ICD-9-CM code 155), pancreatic cancer (ICD-9-CM code 157), lung cancer (ICD-9-CM code 162), melanoma (ICD-9-CM code 172), skin cancers (ICD-9-CM code 173), female breast cancer (ICD-9-CM code 174), male breast cancer (ICD-9-CM code 175), Kaposi’s sarcoma (ICD-9-CM code 176), cervical cancer (ICD-9-CM code 180), uterine cancer (ICD-9-CM code 182), ovarian cancer (ICD-9-CM code 183), prostate cancer (ICD-9-CM code 185), bladder cancer (ICD-9-CM code 188), kidney cancer (ICD-9-CM code 189.0), unspecified urinary organ cancer (ICD-9-CM code 189 excluding 189.0), thyroid cancer (ICD-9-CM code 193), Hodgkin’s lymphoma (ICD-9-CM code 201) and non-Hodgkin’s lymphoma (ICD-9-CM code 202). A cancer was defined as ≥ 2 diagnosis in outpatient visits or inpatient visits. Comorbidities included diabetes mellitus (ICD-9-CM code 250), liver cirrhosis (ICD-9-CM code 571.5) and chronic pancreatitis (ICD-9-CM code 577.1).

### Statistical analyses

Categorical variables were presented as numbers and percentages while continuous variables were reported as means ± standard deviations (SDs). Categorical variables were compared with Chi-square test, and continuous variables were compared with Student’s t test, as appropriate. We first used logistic regression to explore the association of keloid with different kind of cancers. In order to increase the sensitivity to detect this association, the timely association were initially overlooked. After the association of ALL cancers with keloids were successfully established by logistic regression, a cohort study was performed to evaluate the risk of the development of specific cancers in patients with keloid from January 1, 1998 to December 31, 2010. In patients who had a specific cancer diagnosis before a keloid diagnosis, temporality was corrected, and the patients were excluded. Relative risk (RR) of cancer was determined by Cox proportional hazard regression modeling after adjusting common covariates (age, gender and the residential region). In pancreatic cancer, adjusted RR was assessed after adjusting not only above covariates but also reported comorbidities including diabetes mellitus, liver cirrhosis and chronic pancreatitis^10,11^. A two-tailed p-value < 0.05 was considered statistically significant for all analyses. Statistical analyses were performed using SAS version 9.4 (SAS Institute, Cary, NC, USA).
